# Supplementary figures and images for: Essential role of multi-element data in interpreting elevated element concentrations in areas impacted by both natural and anthropogenic influences
Source: PeerJ. 2023 Sep 18;11:e15904. doi: 10.7717/peerj.15904 (PMC10512964; doi:10.7717/peerj.15904)

# Assessment of metal uptake in vegetables under different anthropogenic pressures

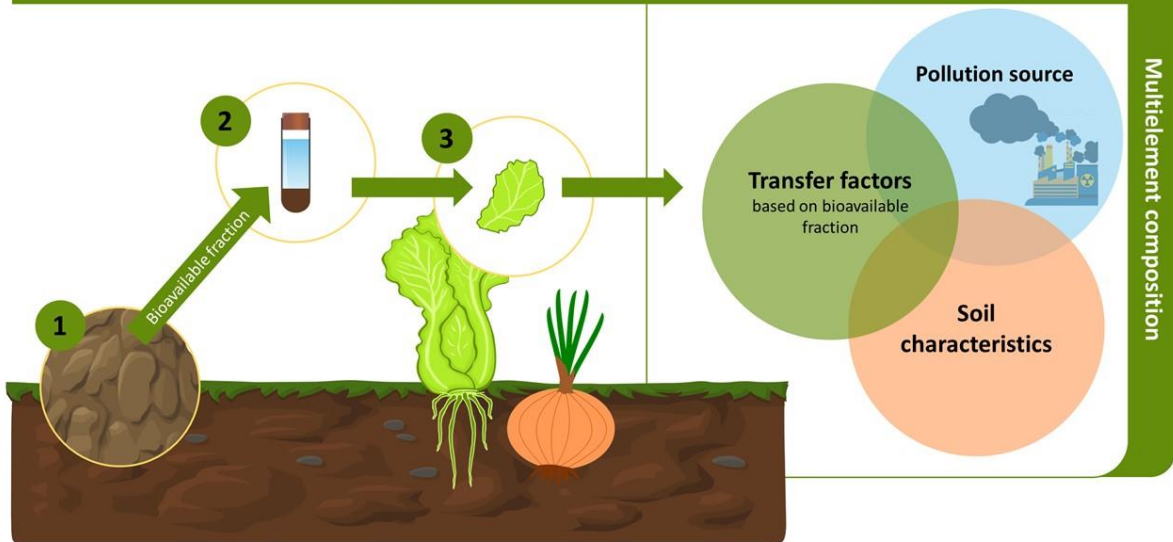

Supplement: Supplemental Information 2 [file peerj-11-15904-s002.pdf]
